# Glycogen Synthase Kinase 3α Is the Main Isoform That Regulates the Transcription Factors Nuclear Factor-Kappa B and cAMP Response Element Binding in Bovine Endothelial Cells Infected with *Staphylococcus aureus*

**DOI:** 10.3389/fimmu.2018.00092

**Published:** 2018-01-29

**Authors:** Octavio Silva-García, Rosa Rico-Mata, María Cristina Maldonado-Pichardo, Alejandro Bravo-Patiño, Juan J. Valdez-Alarcón, Jorge Aguirre-González, Víctor M. Baizabal-Aguirre

**Affiliations:** ^1^Centro Multidisciplinario de Estudios en Biotecnología, Facultad de Medicina Veterinaria y Zootecnia, Universidad Michoacana de San Nicolás de Hidalgo, Morelia, Mexico; ^2^Global Health de México S. A. de C. V., Guadalajara, Mexico

**Keywords:** inflammatory response, endothelial cells, *Staphylococcus aureus*, glycogen synthase kinase 3, nuclear factor-kappa B, cAMP response element binding, interleukin-8, interleukin-10

## Abstract

Glycogen synthase kinase 3 (GSK3) is a constitutive enzyme implicated in the regulation of cytokine expression and the inflammatory response during bacterial infections. Mammals have two GSK3 isoforms named GSK3α and GSK3β that plays different but often overlapping functions. Although the role of GSK3β in cytokine regulation during the inflammatory response caused by bacteria is well described, GSK3α has not been found to participate in this process. Therefore, we tested if GSK3α may act as a regulatory isoform in the cytokine expression by bovine endothelial cells infected with *Staphylococcus aureus* because this bacterium is one of the major pathogens that cause tissue damage associated with inflammatory dysfunction. Interestingly, although both isoforms were phosphorylated–inactivated, we consistently observed a higher phosphorylation of GSK3α at Ser21 than that of GSK3β at Ser9 after bacterial challenge. During a temporal course of infection, we characterized a molecular switch from pro-inflammatory cytokine expression (IL-8), promoted by nuclear factor-kappa B (NF-κB), at an early stage (2 h) to an anti-inflammatory cytokine expression (IL-10), promoted by cAMP response element binding (CREB), at a later stage (6 h). We observed an indirect effect of GSK3α activity on NF-κB activation that resulted in a low phosphorylation of CREB at Ser133, a decreased interaction between CREB and the co-activator CREB-binding protein (CBP), and a lower expression level of IL-10. Gene silencing of GSK3α and GSK3β with siRNA indicated that GSK3α knockout promoted the interaction between CREB and CBP that, in turn, increased the expression of IL-10, reduced the interaction of NF-κB with CBP, and reduced the expression of IL-8. These results indicate that GSK3α functions as the primary isoform that regulates the expression of IL-10 in endothelial cells infected with *S. aureus*.

## Introduction

*Staphylococcus aureus* is a Gram (+) bacterium that causes important infectious diseases among humans and animals. This bacterium expresses a broad range of virulence factors and cell-wall-associated structures (CWASs) that induce inflammation and are responsible for tissue damage (1). A hallmark of *S. aureus* is its ability to evade host innate immune response, which results in repeated infections and life-threatening diseases (2, 3). The molecular mechanisms that *S. aureus* employs to evade the host immune response are diverse, and some of them are poorly understood. One of these mechanisms involves an impairment in cell-signaling pathways leading to the suppression of interleukin-8 (IL-8) expression (4–7), a cytokine that shows pro-inflammatory and chemotactic activities in the first stages of *S. aureus* infections (8, 9). Moreover, IL-8 is essential to promote neutrophil survival and bacterial clearance because the inhibition of IL-8 expression in *S. aureus* infection models has been associated with cell death and bacterial survival (6). *S. aureus* infections also enhance an anti-inflammatory response through the induction of immune-suppressive and -tolerogenic cytokines expression (i.e., interleukin-10 (IL-10)) that contribute to bacterial persistence and immune tolerance (10, 11).

Host cells can recognize *S. aureus* CWASs through the plasma membrane TLR2 receptor. The binding of CWAS to TLR2 activates the phosphoinositide 3-kinase/Akt (PI3K/Akt)-signaling pathway that mediates a variety of cellular responses such as survival, proliferation, differentiation, apoptosis, and inflammation (12). The activation of PI3K leads to Akt phosphorylation at Ser473 and Thr308 by the constitutively active PDK1 and mTORC2 (13). In turn, the activated Akt regulates the activity of a wide range of substrates, among which glycogen synthase kinase 3 (GSK3) regulates the balance of the inflammatory response (14). GSK3 refers to the two mammalian paralogs GSK3α and GSK3β (15), which are unusually constitutively active and can be inactivated by phosphorylation at Ser21 (GSK3α) or Ser9 (GSK3β) by Akt upon bacterial stimulus (16). Since its discovery, GSK3β has been proposed as the principal GSK3 isoform involved in the regulation of many cellular functions, including the inflammatory response caused by bacterial infections. The other isoform, GSK3α, has not yet been associated with the regulation of the inflammatory response. This observation is important because the same stimulus can usually phosphorylate and inactivate both isoforms. The experimental evidence suggests that the physiological roles of GSK3 isoforms may be explained by different regulatory mechanisms, such as the formation of molecular complexes, subcellular compartmentation, and specific kinase modifications that are linked to specific stimuli and cellular contexts (17).

In a previous report, we demonstrated that the internalization of *S. aureus* by bovine endothelial cells (BECs) induced the activity of PI3K/Akt signaling and a preferential phosphorylation of GSK3α compared to GSK3β (18). More recently, we reported that peptidoglycan from *S. aureus* induced a higher phosphorylation of GSK3α than that of GSK3β, and this led to an increase in IL-12p40 expression (19). Several, studies in murine models have shown that both isoforms of GSK3 are not physiologically redundant (20). The deletion of GSK3α in myeloid cells attenuated atherosclerosis and promoted M2 macrophage phenotype and IL-10 expression (21). Interestingly, GSK3 isoforms are differentially expressed, depending on the tissue analyzed. For example, GSK3α is the main isoform expressed in the skeletal muscle, testis, and neutrophils but it is absent in birds (17). Regarding GSK3β, this isoform is predominant in a Th17 subtype of T cells compared to GSK3α, indicating that GSK3α and GSK3β expressions are tissue-specific (22). Also, GSK3α but not GSK3β promotes IL-10 expression in a mice model of high-fat diet-low-density lipoprotein in response to a variety of stimuli (20, 23), and it is the main isoform inactivated by the peptide neurotensin (24), which reduces inflammation after fibroblast stimulation with lipopolysaccharide (LPS) (25).

A molecular mechanism by which GSK3 regulates the balance in cytokines expression has been previously described in macrophages stimulated with LPS from *Escherichia coli*. It was observed that GSK3β regulated the pro- and anti-inflammatory cytokines expression through its influence on nuclear factor-kappa B (NF-κB) and cAMP response element-binding (CREB) activity (14). This mechanism involved the competition between CREB and NF-κB for the co-activator CREB-binding protein (CBP). According to this model, the activation of NF-κB is essential for IL-8 expression (26) and is positively favored by GSK3β (27). By contrast, GSK3β negatively regulates CREB by limiting its phosphorylation at Ser133 (14, 28), which results in a reduced CREB-DNA-binding activity (29) and subsequently low IL-10 expression (30). However, our experimental evidence indicates that this GSK3-dependent regulation of NF-κB and CREB activity in BEC infected with *S. aureus* primarily depends on GSK3α rather than on GSK3β.

Therefore, we propose that in BEC infected with *S. aureus*, the constitutive activity of GSK3α favors NF-κB activation and expression of IL-8 at initial stages. However, GSK3α activity inhibition at later stages of infection favors CREB activation that leads to IL-10 expression. Our data highlight the regulatory role of the GSK3α isoform on the inflammatory response in endothelial cells and may explain, in part, why *S. aureus* infections can evolve to a chronic state.

## Materials and Methods

### Media and Chemicals

DMEM/F12 (F12), Trypsin-EDTA, Igepal CA-930, Akt-IV, and SB216763 were purchased from Sigma–Aldrich, Inc. (St. Louis, MO, USA). Fetal calf serum (FCS) was acquired from Equitech-Bio, Inc. (Kerrville, TX, USA). A cocktail of sodium penicillin G, streptomycin sulfate, and amphotericin B was purchased from Gibco-BRL (Gaithersburg, MD, USA). Halt-TM Phosphatase inhibitor cocktail was purchased from Thermo Fisher Scientific (Waltham, MA, USA). Protease inhibitor cocktail was acquired from GE Healthcare Biosciences (Little Chalfont, UK). Trizol reagent and EXPRESS One-Step SYBR GreenER Universal Kit were purchased from Invitrogen (Carlsbad, CA, USA). Bovine IL-10 and IL-8 TSZ ELISA kit were purchased from Biotang (Waltham, MA, USA). Plasmid pLKO.1-GSK3β-#1 was a gift from Alex Toker (Addgene plasmid #32497) and PLKO.1 plasmid was a gift from Bob Weinberg (Addgene plasmid # 8453). pCF CREB M1 was a gift from Marc Montminy (Addgene plasmid # 22969). The generation of pCF empty vector was done by the enzyme digestion of pCF CREB M1 with SacI restriction enzyme, and then the purification and ligation of the 5-kb fragment were generated.

### Antibodies

Antibodies against Laminin A/C, GAPDH, β-actin, CREB, CBP, and IgG-HRP-coupled antibodies were purchased from Santa Cruz Biotechnology, Inc. (Santa Cruz, CA, USA). Antibodies against phospho-glycogen synthase (Ser641), phospho-GSK3α (Ser21), phospho-GSK3α (Ser9), phospho-NF-κB p65 (Ser536), phospho-CREB (Ser133), GSK3β, GSK3α, and NF-κB p65 were purchased from Cell Signaling Technology (Boston, MA, USA).

### Bacterial Strain, Cell Line, and Culture Conditions

The strain of *S. aureus* used in this study is an isolate from a clinical case of bovine mastitis and was obtained from the American-Type Culture Collection (27543). Bacteria were cultured overnight in 3 ml of Luria-Bertani (LB) medium at 37°C with continuous agitation. The inoculum for infection assays was prepared by adding 1 ml of this pre-culture to 49 ml of fresh LB medium and grown at 37°C. This inoculum was added to cells until the culture reached the initial-middle log phase at an optical density of 0.3 at 600 nm and washed with F12 medium.

Endothelial cells used in this study were obtained from bovine umbilical veins and immortalized by transfection with an expression vector containing the E6–E7 oncogenes of human papillomavirus 16 (BVE-E6–E7) (31). These BECs were grown and maintained in F12 supplemented with 10% FCS and a cocktail of sodium penicillin G, streptomycin sulfate and amphotericin B, unless otherwise noted. For pretreatment with inhibitors, BECs were pre-incubated with a medium alone or with 1 µM of Akt-IV, 10 µM of SB216763, or 20 µM of Parthenolide before infection with bacteria.

### Infection Assay

Bovine endothelial cells were grown in six-well cell culture plates with F12 medium supplemented with FCS and antibiotics until 90–95% confluence. Then, cells were washed 2× with PBS and the medium was changed to F12 without serum and antibiotics. Cells were left in these conditions for at least 4 h. Bacteria were added at a multiplicity of infection (MOI) of 20 CFU/cell, and plates were centrifuged at 500 × *g* for 5 min and incubated in 5% CO_2_ at 37°C for the times indicated.

### BEC Transfection

For plasmids transfection containing siRNA against GSK3 isoforms, BECs were grown until 60–70% confluence in F12 medium without serum and antibiotics for 24 h. Then, transfection was performed with Lipofectamine 2000 from Thermo Fisher Scientific (Waltham, MA, USA); for siGSK3α, we added 3 µg of siRNA-GSK3α plasmid (Sigma–Aldrich) and 3 µg of PLKO.1; for siGSK3β, we added 3 µg of pLKO.1-GSK3β-#1 and 3 µg of PLKO.1; for siGSK3αβ, we added 3 µg of siRNA-GSK3α and 3 µg of pLKO.1-GSK3β-#1 plasmid; and for sicontrol, we added 6 µg of PLKO.1. After 24 h, the culture medium was changed to F12 with low serum (1%), and cells were incubated for 5 days. GSK3α and GSK3β gene-silenced cells were screened by Western blotting. For GSK3α, the targeting sequence was 5′-TACATCTGTTCTCGCTACTA-3′ (nucleotides 1535–1554, pLKO.1-GSK3α), and for GSK3β the targeting sequence was 5′-GAAGTCAGCTATACAGACACT-3′ (nucleotides 587–607, pLKO.1-GSK3β). To express the CREB-dominant-negative mutant, cells were cultured at 70–90% confluence in F12 medium without serum or antibiotics for 24 h; then, 350 ng of plasmid pCF-M1-CREB was transfected with Lipofectamine 2000 or 350 ng of pCF empty vector. pCF-M1-CREB plasmid encodes a CREB mutant in which Ser133 was replaced by Ala133. After 12 h, the medium was changed to fresh F12 and low serum, and cells were incubated for an extended period of 48 h. The medium was changed to F12 without serum or antibiotics, and cells were left for at least 4 h before stimulation with *S. aureus*. Transfection efficiency was monitored by detecting the FLAG tag present on the CREB-mutant construct in Western blot assays. The transfection efficiency of the empty pCF-M1 vector was monitored by the resistance of cells to Geneticin.

### Protein Extraction, Subcellular Fractionation, and Western Blot Assay

To evaluate the relative abundance of phosphorylated and non-phosphorylated proteins, the total protein (cytosolic and nuclear) from control and treated cells was obtained by washing cells 2× with ice-cold PBS and lysing them with 80 µl of cold lysis buffer (20 mM Tris–HCl, pH 7.5, 150 mM NaCl, 1% Igepal CA-930, 10 mM Na-pyrophosphate, 50 mM NaF, and 1 mM Na-orthovanadate) supplemented with 1× protease inhibitor cocktail and 1× phosphatase inhibitor cocktail, which were added immediately before lysis. For subcellular protein fractionation, we followed the REAP method described by Suzuky et al. (32). Lysates were centrifuged at 16,000 × *g* for 20 min at 4°C, and the supernatant was recovered. Protein concentration was measured by Bradford using BSA as standard. Then, 30–40 µg of protein was separated by electrophoresis in 10% sodium dodecyl sulfate-polyacrylamide gels at 200 V for 1 h and electroblotted in a wet chamber onto 0.45 µm nitrocellulose membrane (Bio-Rad) at 250 mA for 1 h. Membranes were incubated in a blocking solution (5% non-fat milk solution) in TTBS (10 mM Tris–HCl, pH 7.5, 0.9% NaCl, and 0.1% Tween 20); primary antibodies to target proteins were added and membranes incubated at 4°C overnight. Membranes washed 1× with PBS and 2× with TTBS were incubated with secondary antibodies. Proteins were detected with the Immobilon Western Chemiluminescent HRP substrate kit from Millipore (Billerica, MA, USA). Protein loading was verified by stripping the membranes with NaOH of 0.2 N for 5 min and washed 2× with TTBS; after a blocking step, the membranes were re-probed with antibodies against the indicated housekeeping proteins and detected in the same way as described. Data presented in Figures 4 and 9C were obtained by scanning the membranes using a C-DiGit blot scanner (LI-COR, Lincoln, NE, USA).

### RNA Extraction and Reverse Transcription and Real-time Quantitative PCR (qRT-PCR)

After infection, BECs were washed 2× with ice-cold PBS, and the total RNA was extracted with Trizol reagent, following the isolation procedure described by the manufacturer. RNA was purified with RQ1 RNase-free DNase (Promega). One-step qRT-PCR was performed using the EXPRESS One-Step SYBR GreenER Universal Kit and the real-time StepOnePlus thermocycler from Applied Biosystems. Each reaction was performed with 100 ng of total RNA under the standard 20-µl reaction provided by Invitrogen. The one-step-cycling program conditions and oligonucleotide primers used were as described by Konnai et al. (2003) (33). Amplification of the expected single products was confirmed by single-peak analysis in temperature-melting curve and visualization on 1% agarose gels stained with ethidium bromide. Relative transcript levels of mRNA were calculated with the ΔΔCt method, using β-actin as the reference gene. Negative amplification of non-added RT enzyme reactions confirmed the absence of DNA in our assays.

### Measurement of IL-8 and IL-10 Protein Levels

Bovine IL-8 and IL-10 proteins in culture supernatants were quantitated for the indicated times by sandwich ELISA, according to the manufacturer’s instructions (Biotang, Waltham, MA, USA).

### Co-IP Assays

Co-IP assays were performed with the Pierce Protein A/G Magnetic Beads kit (Thermo Fisher Scientific). Briefly, the control and infected cells (~1 × 10^7^) were washed 2× with ice-cold PBS and lysed in washing/lysis buffer supplemented with 1× protease inhibitor cocktail and 1× phosphatase inhibitor cocktail. Cell debris was collected by centrifugation at 18,000 × *g* for 10 min at 4°C. The supernatant was recovered and quantitated for protein concentration. Then, 5 µg of CBP IP antibody was added to 500 µg of protein extract; the volume was adjusted to 500 µl with lysis buffer and incubated with continuous agitation overnight at 4°C. Then, 25 µg of protein A/G magnetic beads was added and incubated for 1 h with continuous agitation at room temperature (~23°C); beads were washed 2× with a washing buffer and 1× with water. The target antigen was eluted with 1× lane marker buffer and 50 mM DTT in a final volume of 100 µl. Then, 30 µl of sample in elution buffer was loaded, and proteins were detected by Western blot as described earlier.

### Statistical Analysis

The relative abundance of phosphorylated proteins was quantitated by densitometric analysis with the Image Processing and Analysis in Java Program ImageJ (http://rsbweb.nih.gov/ij). To calculate densitometric values, the intensity of the phosphorylated band was divided by the intensity of the non-phosphorylated one. These intensities were referred to a value of 1.0 that was arbitrarily assigned to the untreated control. The statistical significance of triplicate blots, IL-8 and IL-10 mRNA ΔΔCt value, and protein levels were evaluated with the Tukey HSD multiple-comparison test and one-way analysis of variance by using the JMP 6.0 program (SAS Institute). Differences between groups were considered significant at *P* < 0.05. All data are available on request.

## Results

### Endothelial Cells Infected with *S. aureus* Express IL-8 and IL-10

**Figure 1:**
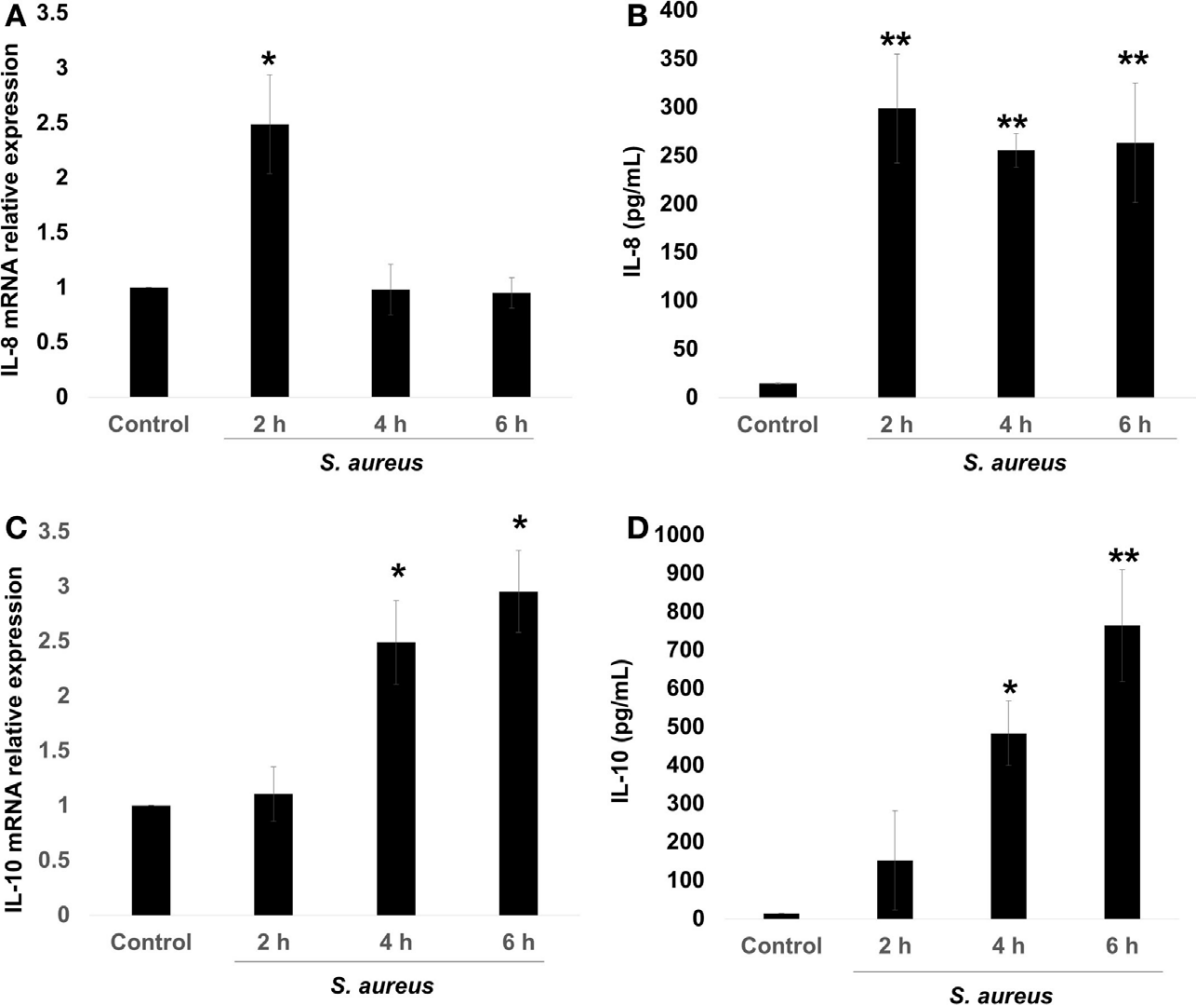
*Staphylococcus aureus* infection induces interleukin-8 (IL-8) and interleukin-10 (IL-10) expressions in bovine endothelial cells (BECs). BEC controls were left uninfected or infected at a multiplicity of infection of 20 CFU/cell *S. aureus* for 2–6 h. Then, the total RNA was extracted and the relative expression of IL-8 mRNA **(A)** and IL-10 mRNA **(C)** was quantified by reverse transcription and real-time quantitative PCR using the ΔΔCt method. Data were normalized in reference to the expression of β-actin. Quantitation of IL-8 **(B)** and IL-10 **(D)** peptides was done by ELISA. Data are presented as the mean ± SEM of three independent experiments (SEM; *n* = 3 per experiment). *P < 0.05, **P < 0.01 compared to control.

The pro-inflammatory chemokine IL-8 is an early marker of bacterial infection (9). However, several authors have reported that *S. aureus* can induce or repress the IL-8 expression on different cellular physiological contexts, and this was correlated with the pathology produced by this bacterium (4–7). There is also evidence that *S. aureus* induces IL-10 expression, leading to immune tolerance (11). Therefore, we asked whether the expression of these cytokines depended upon the stage of infection. In order to evaluate the kinetics of IL-8 and IL-10 expressions, BECs were infected with *S. aureus* at a MOI of 20, which allows the survival of endothelial cells for 2, 4, and 6 h. Then, RNA was purified to analyze IL-8/IL-10 mRNA by qRT-PCR and their corresponding peptides in cell supernatants by ELISA. The expression of IL-8 mRNA increased by more than 2.5-fold in response to 2 h of *S. aureus* infection; however, at longer infection times of 4–6 h, IL-8 mRNA values returned to control levels (Figure 1A). Measurements of IL-8 peptide indicated an increase at 2 h post infection with similar values at 4 and 6 h (Figure 1B). In the case of IL-10 mRNA, an increase was observed at 4 h and remained without a statistical change for up to 6 h (Figure 1C) while its peptide reached a maximum at 4 h (Figure 1D). These data indicate that in BEC infected with *S. aureus*, the mechanism responsible for IL-8 expression precedes the mechanism leading to IL-10 expression.

### *S. aureus* Infection Induces a Time-Dependent NF-κB/CREB Phosphorylation and NF-κB Nuclear Translocation

**Figure 2:**
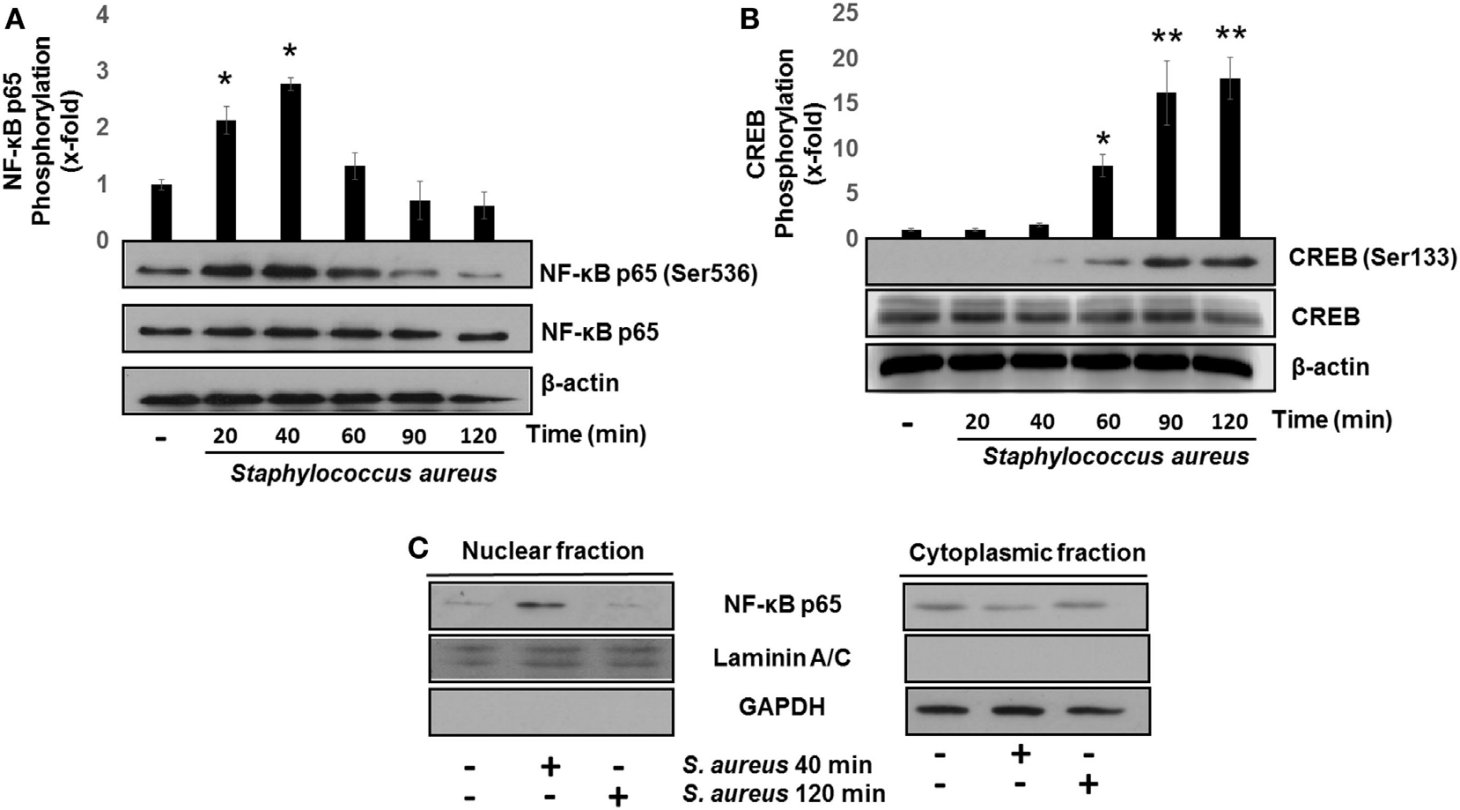
*Staphylococcus aureu*s activates nuclear factor-kappa B (NF-κB) and cAMP response element-binding (CREB) phosphorylation and induces NF-κB nuclear translocation. Bovine endothelial cells were left uninfected (−) or infected at a multiplicity of infection of 20 CFU/cell of *S. aureus* for 20–120 min. After infection, the total protein (nuclear + cytoplasmic) of **(A,B)** or nuclear- and cytoplasmic-enriched fractions **(C)** was analyzed by Western blotting to detect the relative abundance of phosphorylated NF-κB p65 at Ser536 **(A)**, phosphorylated CREB at Ser133 **(B)**, or NF-κB p65 **(C)**. The detection of total unphosphorylated NF-κB p65 **(A)**, total unphosphorylated CREB **(B)**, and β-actin **(A,B)** was performed to ensure equal protein loading. GAPDH and Laminin A/C were detected as protein markers to ensure equal protein loading for cytoplasmic- and nuclear-enriched fractions, respectively **(C)**. Error bars in graphs A and B represent the mean ± SEM (*n* = 3) of densitometric values. *P < 0.05, **P < 0.01 referred to the uninfected control value.

The transcription factors NF-κB and CREB induce IL-8 and IL-10 expressions, respectively (26, 34). The transcriptional activation of NF-κB, in response to bacterial ligands, involves the phosphorylation of p65 subunit at Ser536 and its translocation to the nucleus. These events usually take place in the first 15–40 min after cell stimulation with different ligands (8, 14, 32, 35, 36). We observed that the phosphorylation of p65 at Ser536 reached a maximum level at 40 min after bacterial challenge followed by a gradual reduction as compared to uninfected control (Figure 2A). Several authors have demonstrated that CREB is transcriptionally activated by phosphorylation at Ser133 in response to various stimuli (28, 37, 38). Accordingly, a significant increase in phospho-CREB at Ser133 at 60 min post infection was detected with a maximum observed at longer infection times of 90–120 min (Figure 2B). Interestingly, these longer infection times that we tested to induce an increase in phospho-CREB-Ser133 were the same as those required for NF-κB to become dephosphorylated and inactivated. These results allowed us to select 40 and 120 min as the infection times to explore the effects of *S. aureus* infection on NF-κB p65 and CREB, respectively.

The transcription of NF-κB-specific target genes involves its translocation from the cytoplasm to the nucleus. To evaluate NF-κB nuclear translocation, we infected BEC with *S. aureus* at 40 and 120 min and detected the relative abundance of NF-κB p65 subunit present in the nucleus or cytosol by Western blotting. The NF-κB p65 subunit was detected in the nuclear fraction after 40 min of infection, whereas at 120 min, we could not observe it (Figure 2C). These indicate that NF-κB phosphorylation and nuclear translocation are processes that take place in the initial phase of infection and precede CREB activation.

### GSK3α/β Inhibition Favors CREB Phosphorylation at Ser133 in BEC Infected with *S. aureus*

**Figure 3:**
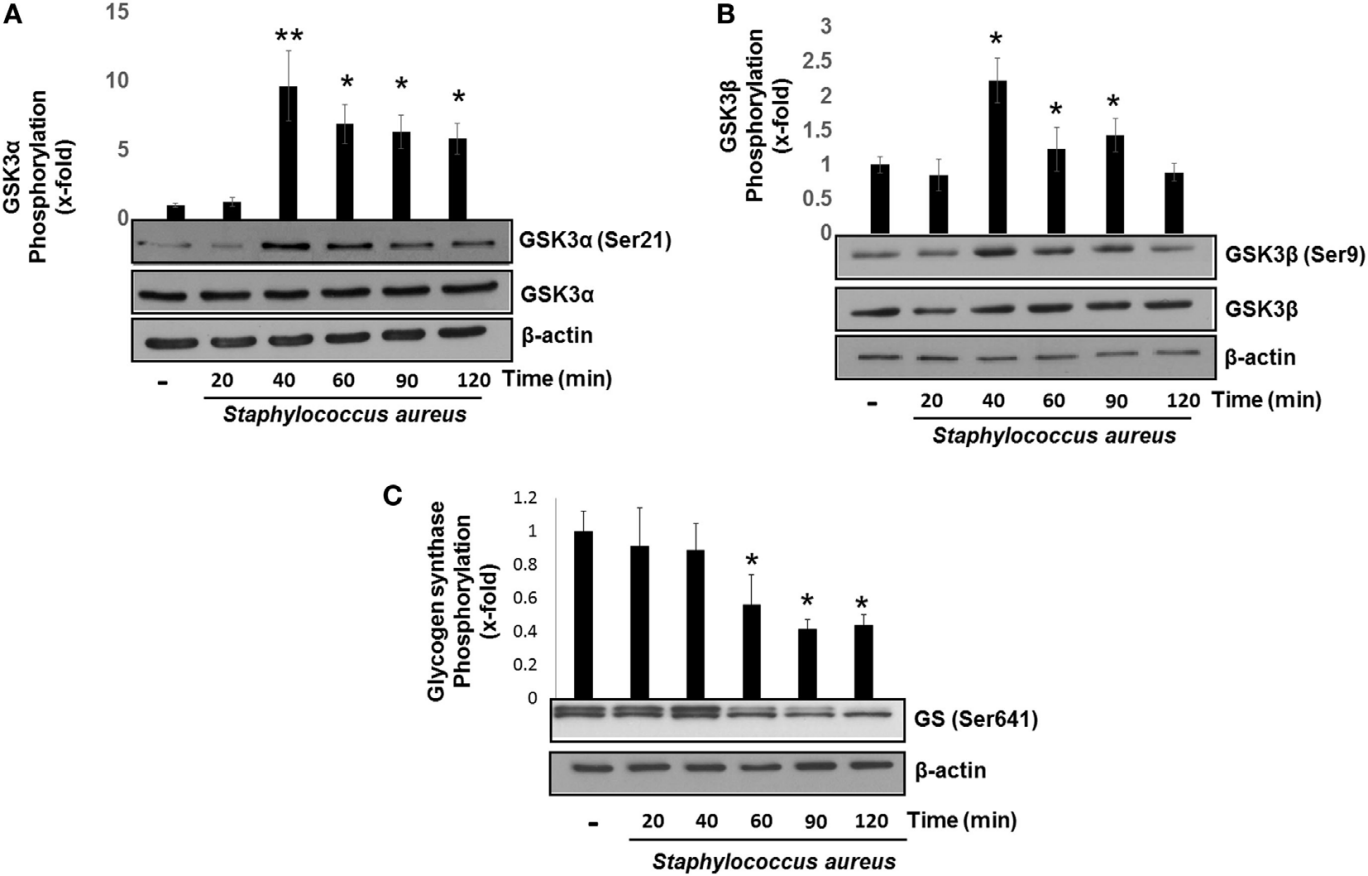
*Staphylococcus aureus* inactivates glycogen synthase kinase 3 (GSK3α) and GSK3β by phosphorylation at Ser21 and Ser9. Bovine endothelial cells were left uninfected (−) or infected at a multiplicity of infection of 20 CFU/cell of *S. aureus* for 20–120 min **(A,B)** or 20–60 min **(C)**. The relative abundance of phosphorylated GSK3α at Ser21 **(A)**, GSK3β at Ser9 **(B)**, and glycogen synthase at Ser641 **(C)** was evaluated by Western blotting. The detection of total GSK3α, GSK3β, and β-actin was done to ensure equal protein loading. Error bars in graphs A–C are presented as the mean ± SEM of three independent experiments (SEM; *n* = 3 per experiment). *P < 0.05, **P < 0.01 as compared to control.

GSK3α and GSK3β are constitutive active enzymes that regulate the activity of several transcription factors (15, 39). We have previously reported that PI3K/Akt-signaling pathway leads to the phosphorylation of GSK3α at Ser21 and GSK3β at Ser9 in *S. aureus* internalization by BEC (18). However, the role of each isoform in cytokine regulation was not evaluated. Therefore, we decided to investigate the contribution of GSK3α and GSK3β to the regulation of NF-κB and CREB activation in BEC infected with *S. aureus*. The time course of BEC infected with *S. aureus* showed that the phosphorylation of GSK3α (Ser21) and GSK3β (Ser9) increased significantly after 40 min of infection (Figures 3A,B), GSK3α phosphorylation (~8-fold compared with control) being higher than GSK3β (~2.5-fold compared with control). Interestingly, under basal conditions, BEC phosphorylation of GSK3α Ser21 is barely detected. Other authors have reported similar results about GSK3 relative phosphorylation under basal conditions in different cell types (40–43).

**Figure 4:**
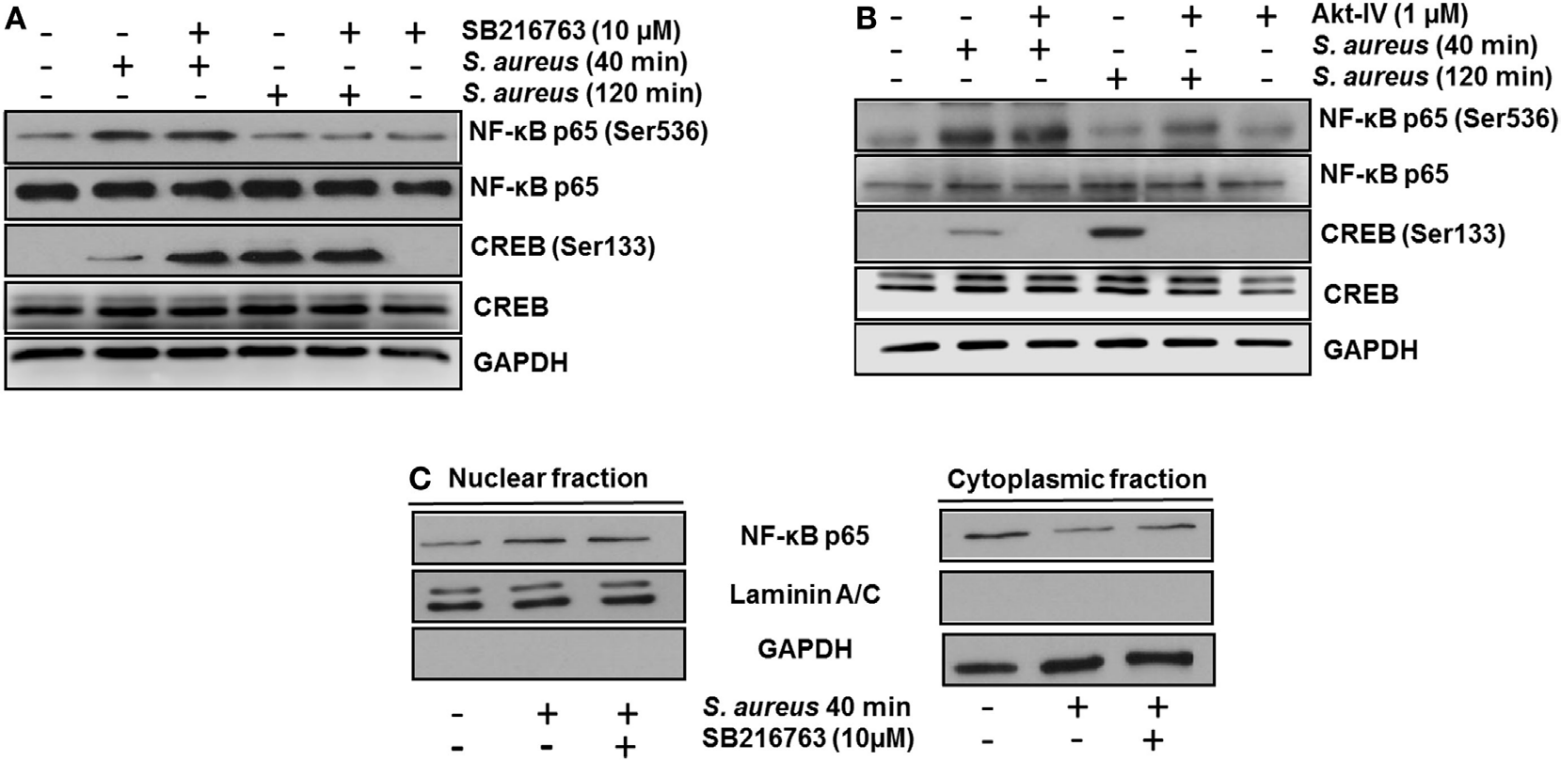
The constitutive activity of glycogen synthase kinase 3 (GSK3α/β) isoforms regulates nuclear factor-kappa B (NF-κB) and cAMP response element-binding (CREB) activity. Bovine endothelial cells (BECs) were left untreated and uninfected (−) or pretreated with the GSK3α/β inhibitor SB216763 (10 µM) **(A)** or the Akt inhibitor Akt-IV (1 µM) **(B)** for 1 h before infection at a multiplicity of infection of 20 CFU/cell of *Staphylococcus aureus* for the indicated times. Controls pretreated with the inhibitors alone but not infected were also included. After infection, the relative abundance of phosphorylated forms of NF-κB p65 at Ser536 and CREB at Ser133 was detected by Western blotting. Unphosphorylated NF-κB p65 and CREB, and GAPDH were detected to ensure equal protein loading. **(C)** BECs were left untreated and uninfected (−) or pretreated with the GSK3α/β inhibitor SB216763 (10 µM) for 1 h before infection with 20 CFU/cell of *S. aureus* for the indicated time. After infection, nuclear- and cytoplasmic-enriched fractions were obtained, and unphosphorylated NF-κB p65 was detected. GAPDH and Laminin A/C were detected as protein markers to ensure equal protein loading for cytoplasmic- and nuclear-enriched fractions, respectively. Blots are representative of three independent experiments.

Next, we analyzed whether the increase in the relative abundance of phosphorylated GSK3α and GSK3β isoforms correlated with a decrease in the phosphorylation of glycogen synthase at Ser641 in BEC infected with *S. aureus*. A marked reduction of GSK3α/β kinase activity from 60 to 120 min post infection led to a gradual decrease in the relative abundance of phosphorylated glycogen synthase at Ser641 (Figure 3C). To further explore if GSK3α/β was associated with NF-κB p65 phosphorylation at Ser536, BECs were pretreated with SB to inhibit GSK3α/β activity before infection for 40 and 120 min. The inhibition of GSK3α/β with SB did not reduce the relative abundance of NF-κB p65 phosphorylated at Ser536 (Figure 4A, upper panel). In contrast to NF-κB, CREB phosphorylation was affected by the inhibition of GSK3α/β activity, because in *S. aureus-*infected cells, SB induced a substantial increase in the relative abundance of phospho-CREB-Ser133 compared to infected cells at 40 min (Figure 4A, middle panel). Interestingly, at 120 min, SB did not induce a greater increase in phospho-CREB-Ser133 compared to infected cells, indicating that the maximal increase of phospho-CREB-Ser133 in SB-pretreated cells peaked at 40 min post infection. Because these observations showed that GSK3 inhibition accelerated the phosphorylation of CREB-Ser133, we tested the relative abundance of p65 and CREB phosphorylation in BEC treated with Akt-IV, an Akt inhibitor. We observed that Akt inhibition did not affect NF-κB p65 phosphorylation level at 40 min of infection; however, at 120 min, a slight increase in phospho-p65 was observed, suggesting that GSK3α/β activity promotes NF-κB phosphorylation at later stages of infection (Figure 4B, upper panel). Akt inhibition completely abolished CREB phosphorylation, indicating that the activated state of GSK3α/β inhibits phospho-CREB-Ser133 increase (Figure 4B, middle panel). Finally, the nuclear accumulation of NF-κB p65 was not affected by GSK3α/β inhibition with SB (Figure 4C).

**Figure 5:**
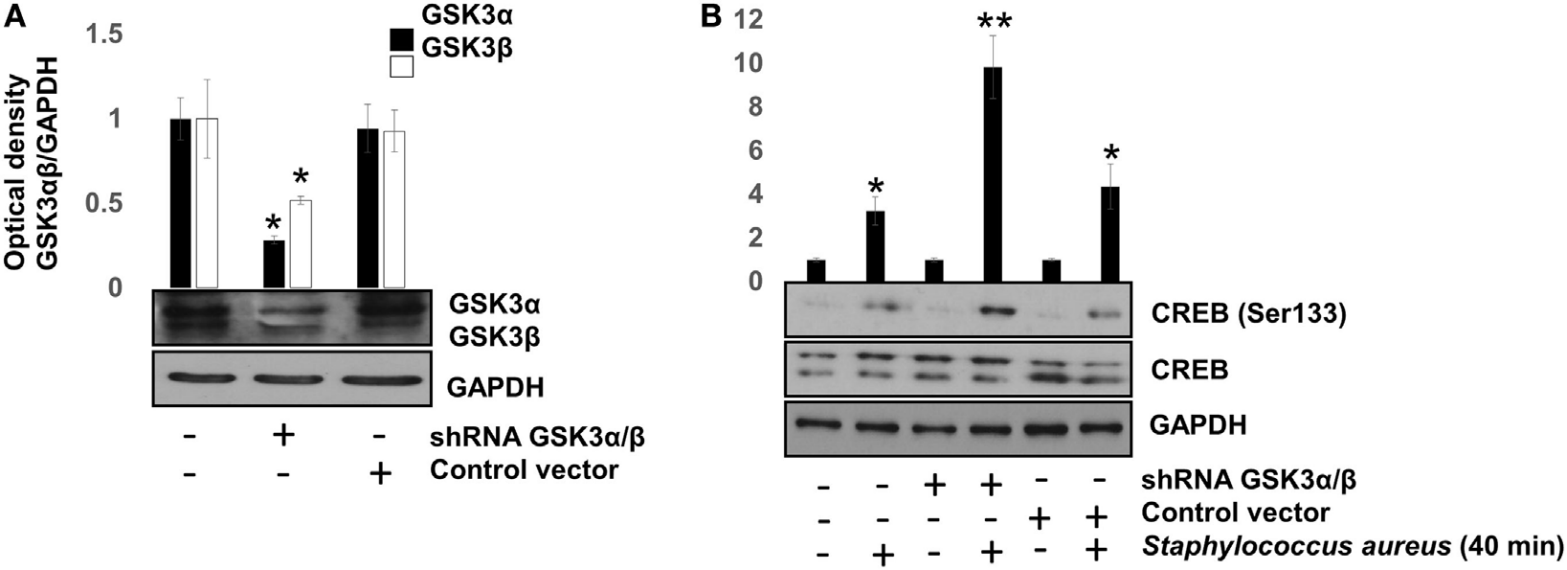
The glycogen synthase kinase 3 (GSK3α/β) isoforms activity inhibits cAMP response element-binding (CREB) phosphorylation at Ser133. Bovine endothelial cells (BECs) were left with medium only or transfected for 120 h with PLKO.1 vector containing siRNA sequences to silence the expression of GSK3α and GSK3β genes or with PLKO.1 control vector. **(A)** Unphosphorylated GSK3α/β was detected to check for protein loading. Error bars in graph represent the mean ± SEM (*n* = 3) of three independent experiments. *P < 0.05, referred to the untransfected control. **(B)** BECs were infected with 20 CFU/cell of *Staphylococcus aureus* for 40 min. After infection, the relative abundance of phosphorylated CREB at Ser133 was analyzed by Western blotting. Unphosphorylated CREB and GAPDH were detected to ensure equal protein loading. Error bars in graph represent the mean ± SEM (*n* = 3) of three independent experiments. *P < 0.05, **P < 0.001 compared to control.

To confirm that GSK3α/β activity interferes with CREB phosphorylation at Ser133, the genes of each GSK3 isoform were siRNA-silenced for 120 h. After this period, we infected BEC with *S. aureus* for 40 min, a time in which GSK3α/β is still active and CREB phosphorylation was barely higher than the uninfected control (Figure 2B). GSK3α/β gene silencing was about 60–70% for GSK3α and 50–60% for GSK3β (Figure 5A), demonstrating the efficiency of the siRNA interference technique. Under these conditions, phospho-CREB-Ser133 showed a marked increase in GSK3α/β-silenced cells, compared to non-transfected or control vector cells (Figure 5B). These data demonstrate that (1) NF-κB p65 phosphorylation at Ser536 and its nuclear translocation are not directly affected by GSK3α/β activity, (2) GSK3α/β activity directly affects CREB phosphorylation at Ser133, and (3) Akt plays a major role in CREB phosphorylation.

### GSK3α Activity Regulates the Interaction between CBP and NF-κB or CREB

**Figure 6:**
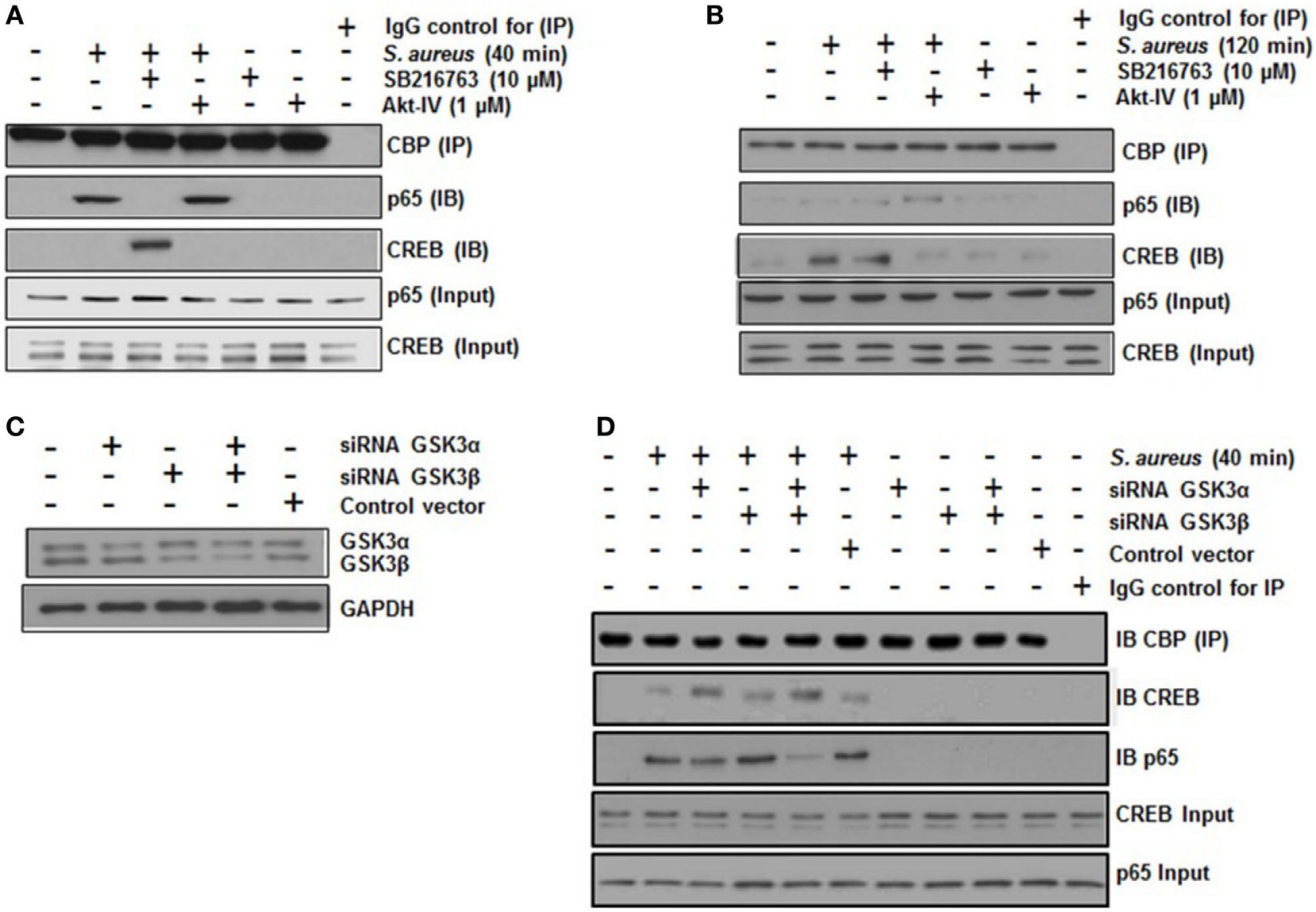
The glycogen synthase kinase 3 (GSK3α/β) isoforms regulate the interaction of nuclear factor-kappa B (NF-κB) and cAMP response element binding (CREB) with the transcriptional co-activator CREB-binding protein (CBP). **(A,B)** Cells were left uninfected and untreated (−) or pretreated with the GSK3α/β inhibitor SB216763 (10 µM) or the Akt inhibitor Akt-IV (1 µM) for 1 h and infected with 20 CFU/cell of *Staphylococcus aureus* for 40 **(A)** or 120 min **(B)**. The total protein was extracted (input fraction) for immunoprecipitation (IP) of CBP. Eluates obtained were then used to detect (IB) NF-κB or CREB by Western blotting. A negative control with an iso-type IgG control was also included. NF-κB and CREB were detected in the input fractions to ensure equal protein loading. **(C,D)** Cells were transfected with plasmids containing siRNA sequences against GSK3α, GSK3β, or with control vector as indicated for 5 days. Then, siRNA of GSK3 isoforms was evaluated by Western blotting. **(D)** GSK3α/β-silenced cells were infected with *S. aureus* for 120 min, and then CBP protein was immunoprecipitated from cell lysates; NF-κB and CREB proteins were detected in the CBP immunoprecipitates by Western blotting. IgG control was included and the total CREB and NF-κB in input cell lysates are also shown.

A critical step in the transcriptional regulation mediated by NF-κB or CREB is the interaction of each of these transcription factors with the co-activator CBP. NF-κB and CREB compete for the limiting amounts of CBP to form a complex in the nucleus (14, 44). To test whether GSK3α/β activity was necessary for NF-κB-CBP or CREB-CBP complex formation in BEC infected with *S. aureus*, we carried out co-IP assays. When BECs were pre-incubated with SB and infected with *S. aureus* for 40 min, IP of CBP showed a marked reduction of the NF-κB-CBP interaction and a strong increase of the CREB-CBP complex (Figure 6A). Accordingly, the inhibition of Akt with Akt-IV (GSK3α/β remains active under this condition) favored NF-κB, but not CREB, interaction with CBP. The inhibition of GSK3α/β with SB at 40 min or control without SB at 120 min led to an increase in CREB-CBP interaction (Figure 6B). Moreover, at 120 min post infection, the Akt inhibition favored the interaction of NF-κB with CBP (Figure 6B). These data suggest that an initial stage of infection GSK3α/β activity indirectly favors NF-κB-CBP complex formation but not CREB-CBP interaction, whereas at later stages of infection, when GSK3α/β loses its kinase activity, the opposite occurs.

To identify which GSK3 isoform was predominantly affecting CREB-CBP or NF-κB-CBP complex formation, we performed an siRNA-silencing assay to independently reduce the expression of each isoform (Figure 6C). After infection of BEC with *S. aureus* for 40 min, we immunoprecipitated CBP, and CREB or NF-κB was immunodetected in the precipitated fraction. In *S. aureus*-infected cells, GSK3α was the primary isoform that affected CREB-CBP interaction, because in either GSK3α or GSK3α/β-silenced cells, a stronger interaction between CREB and CBP was observed compared to the amount of CREB-CBP complex seen in GSK3β-silenced cells (Figure 6D). This preferential association found between CREB and CBP in GSK3α-silenced cells repressed NF-κB-CBP interaction because of the reduced amount of NF-κB-CBP complex in these cells compared to the infected controls or the GSK3β-silenced cells.

### NF-κB and CREB Regulate IL-8 and IL-10 Expressions in BEC Infected with *S. aureus*

**Figure 7:**
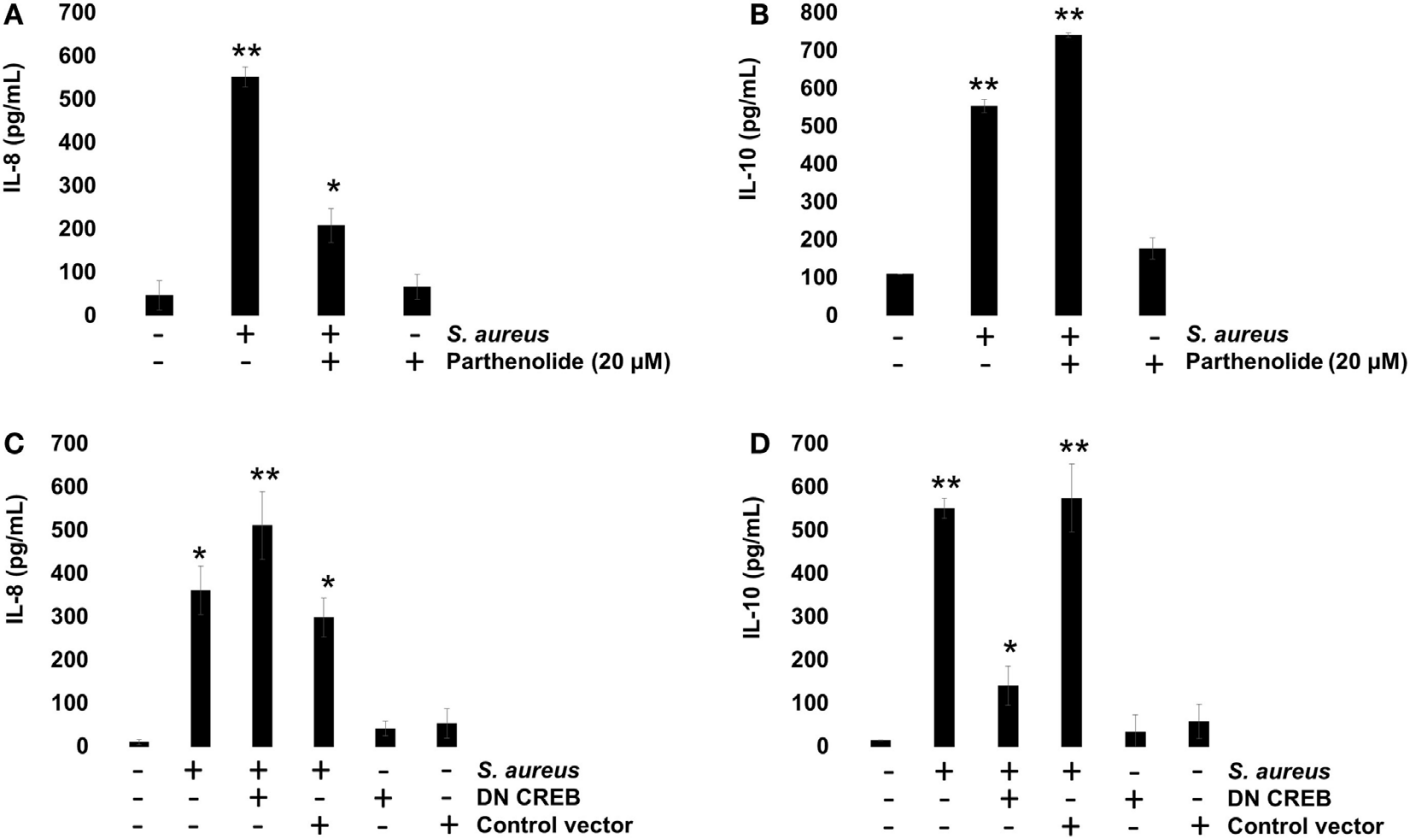
*Staphylococcus aureus*-induced expression of interleukin-8 (IL-8) and interleukin-10 (IL-10) is regulated by nuclear factor-kappa B (NF-κB) and cAMP response element binding (CREB). **(A,B)** Bovine endothelial cells (BECs) were left uninfected and untreated (−) or pretreated with the NF-κB inhibitor Parthenolide (20 µM) for 1 h and then infected with 20 CFU/cell of *S. aureus* for 6 h. A control group pretreated with 20 µM of Parthenolide was also included. **(C,D)** BECs were treated with a medium or transfected with a plasmid containing the dominant-negative mutant of CREB-S133A (DN-CREB) or the control plasmid pCF (control vector) for 48 h and then infected with 20 CFU/cell of *S. aureus* for 6 h. Control-uninfected cells transfected with the DN-CREB or plasmid pCF were included. After infection, the conditioned media were collected in ice-cold tubes and kept frozen at −80°C. Quantitation of IL-8 and IL-10 was done by ELISA, according to the manufacturer’s instructions. Data represent the mean ± SEM (*n* = 3) from two independent experiments. *P < 0.05, **P < 0.01 as compared to control.

To evaluate the role of NF-κB on IL-8 and IL-10 expressions, we incubated BEC with 20 µM Parthenolide, an NF-κB inhibitor, for 1 h before infection. Under these conditions, a reduced IL-8 expression was observed at 2 h post infection (Figure 7A), whereas an increased IL-10 expression was detected at 6 h post infection (Figure 7B), indicating that NF-κB activity causes a slight inhibition of IL-10 expression in BEC infected with *S. aureus*. Then, we evaluated the role of CREB on IL-8 and IL-10 expressions by transfecting BEC with a plasmid containing a CREB-Ser133Ala-dominant-negative mutant (DN-CREB-Ser133Ala) (Figure S1 in Supplementary Material). At 2 h post infection, the DN-CREB-Ser133Ala-expressing cells showed an increased expression of IL-8 (Figure 7C). By contrast, at 6 h post infection, the DN-CREB-Ser133Ala-expressing cells showed a significant reduction of IL-10 expression (Figure 7D). These results confirm that NF-κB and CREB compete for binding CBP and that they are essential for IL-8 and IL-10 expressions in BEC infected with *S. aureus*.

### GSK3α/β Activity Regulates IL-8 and IL-10 Expressions in BEC Infected with *S. aureus*

**Figure 8:**
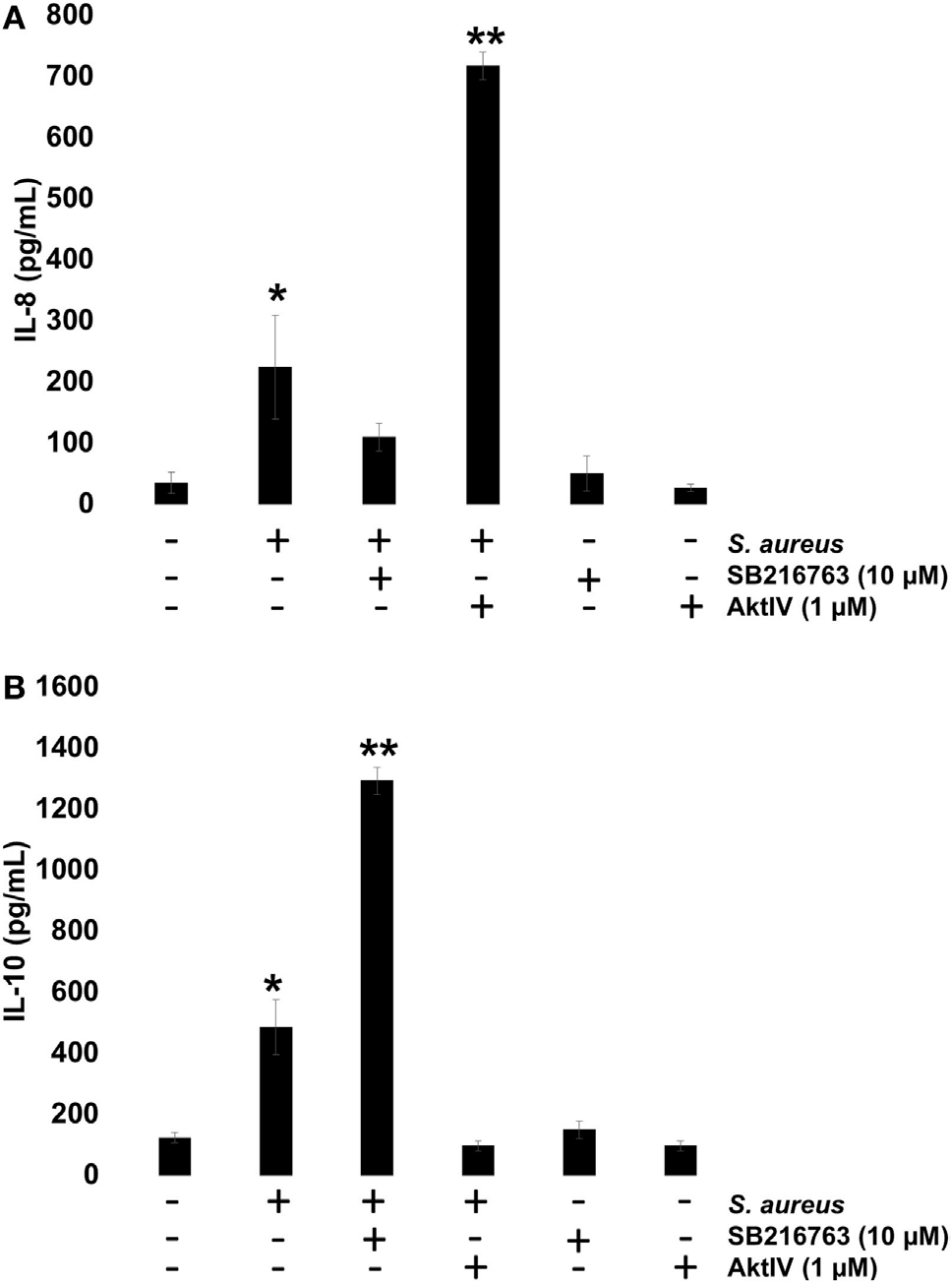
The glycogen synthase kinase 3 (GSK3α/β) activity controls *Staphylococcus aureus*-induced expression of interleukin-8 (IL-8) and interleukin-10 (IL-10). **(A,B)** Bovine endothelial cells (BECs) were left uninfected and untreated (−) or pretreated with GSK3α/β inhibitor SB216763 (10 µM) or Akt inhibitor Akt-IV (1 µM) for 1 h and then infected with 20 CFU/cell of *S. aureus* for 6 h. A control pretreated with SB216763 (10 µM) or Akt-IV (1 µM) was also included. After infection, the conditioned media were collected in ice-cold tubes and kept frozen at −80°C. The quantitation of IL-8 and IL-10 was done by ELISA, according to the manufacturer’s instructions. Data represent the mean ± SEM (*n* = 3) from two independent experiments. *P < 0.05, **P < 0.01 compared to control.

To investigate whether GSK3α/β activity influences NF-κB and CREB transcriptional activity and this, in turn, regulates IL-8 and IL-10 expressions, BECs were pre-incubated with SB or Akt-IV for 1 h. After infection with *S. aureus* for 6 h, the IL-8 and IL-10 peptides were quantitated by ELISA. We observed a reduction in IL-8 peptide compared to the infected control cells in the presence of SB, whereas in BEC treated with Akt-IV, a condition in which GSK3α/β isoforms are active, the IL-8 expression was higher after 6 h as compared to SB treated or infected control cells (Figure 8A). The expression of IL-10 increased when BECs were incubated with SB at 6 h post infection, whereas in the presence of Akt-IV, no change in IL-10 peptide compared to control was observed, indicating that active Akt (inactive GSK3α/β) was required to induce IL-10 synthesis (Figure 8B). These data suggest that GSK3α/β activity promotes IL-8 peptide synthesis during *S. aureus* infection and that the loss of GSK3α/β activity leads to a reduction of IL-8 and an increase in IL-10 expression.

### GSK3α Activity Is the Main Isoform That Regulates IL-8 and IL-10 Expressions in BEC Infected with *S. aureus*

**Figure 9:**
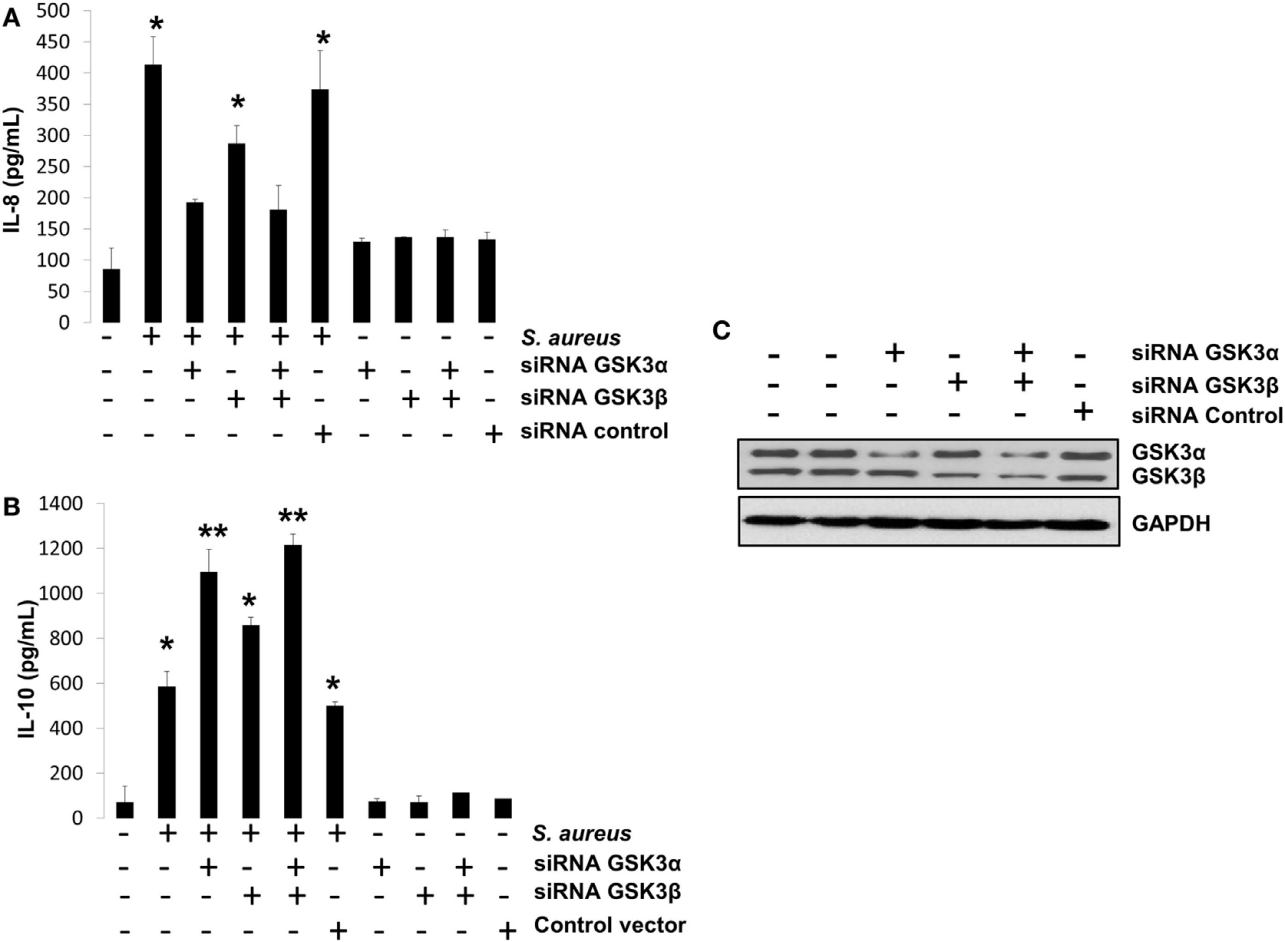
The glycogen synthase kinase 3 (GSK3α) is the primary isoform that regulates interleukin-8 (IL-8) and interleukin-10 (IL-10) expression in bovine endothelial cell infected with *Staphylococcus aureus*. **(A–C)** Cells were left untransfected or transfected with plasmids containing siRNA against GSK3α or GSK3β or empty vector as indicated for 5 days. Then, cells were infected with *S. aureus* at a multiplicity of infection of 20 UFC/cell for 6 h or with a medium alone, and IL-8 **(A)** and IL-10 **(B)** peptide expressions were evaluated by ELISA technique. Controls uninfected/untransfected and transfected/uninfected were also included. **(C)** A Western blot to represent the effectiveness of silencing technique is also shown. Data represent mean ± SEM from results obtained in two independent experiments measured by duplicate. *P < 0.05, **P < 0.01 compared to control.

We have presented evidence that in BEC infected with *S. aureus*, (1) GSK3α is highly phosphorylated, (2) GSK3α promotes CREB-CBP interaction, which is essential for IL-10 expression, and (3) GSK3α represses NF-κB-CBP interaction, which is critically indispensable for IL-8 expression. To confirm that GSK3α is the main isoform regulating these processes, we performed an siRNA-gene-silencing assay to reduce the expression of each GSK3 isoform in BEC infected with *S. aureus* for 6 h followed by the detection of IL-8 and IL-10 peptides by ELISA. We observed that GSK3α was the main isoform that regulates IL-8 and IL-10 expressions because GSK3α or GSK3α/β-silenced cells showed a stronger reduction of IL-8 expression and an increased IL-10 expression compared to GSK3β-silenced or control-infected cells (Figures 9A,B). Evidence of GSK3α and GSK3β gene-silencing efficiency is shown in Figure 9C. These data demonstrate that GSK3α is the primary isoform that regulates the expression of IL-8 and IL-10 in BEC infected with *S. aureus*.

## Discussion

Several reports have described an inhibition of IL-8 expression after a “*short-live*” temporary increase when *S. aureus* infects a variety of cells (4–7, 45). For example, in conjunctiva epithelial cells, a significant increase in IL-8 mRNA was detected at 3 h post infection and a marked reduction after 4 h, an effect attributed to phenol-soluble modulins from *S. aureus* (45). Also, bovine mammary epithelial cells infected with *S. aureus* expressed IL-8 at initial stages (8 h), but at longer infection times (24–48 h), no increase was detected (46). In neutrophils, *S. aureus* strain USA300 inhibited NF-κB-dependent IL-8 expression and promoted cell death (6). Interestingly, in this case, the inhibition of NF-κB was related to the *S. aureus* SaeR/S system regulation, which is important for phenol-soluble modulins expression (47). *S. aureus* infections also promoted IL-10 expression, a cytokine associated with bacterial survival and immune-tolerogenic response (10, 11, 48). Interestingly, as it is the case for IL-8, the phenol-soluble modulins were also involved in IL-10 expression by dendritic cells (49). The expression of IL-6 and TNF-α at initial stages of infection and IL-10 at longer stages was also detected in the serum of mice infected with *S. aureus* (50); however, in all these reports, a molecular mechanism was not described. In this work, we have presented experimental evidence indicating that BEC infected with *S. aureus* activates NF-κB and expresses IL-8 at initial stages of infection (2 h) and activates CREB and IL-10 expression at later stages (6 h). Apparently, this molecular switch from pro-inflammatory to an anti-inflammatory cytokine expression is mainly regulated by the activity state of GSK3α. We have also shown that GSK3α regulates the balance of cytokines expression by modulating the activity of NF-κB and CREB in a similar way as GSK3β (14).

For more than 10 years, GSK3β has been considered the main isoform responsible for the regulation of the inflammatory response (14, 51). Various studies have confirmed that GSK3β regulates the response to infections caused by Gram (−)/(+) bacteria like *Helicobacter pylori, Francisella tularensis*, and *Mycobacterium* sp. (52–54). Although several reports have provided evidence that GSK3α is the main isoform expressed in various cell types, shares the same substrates, and is also inactivated in similar ways as GSK3β (55–57), no correlation of GSK3α activity on the regulation of the inflammatory response after bacterial stimulation has been documented. This lack of information prompted us to explore whether GSK3α may be responsible for the regulation of pro- and anti-inflammatory cytokines expression in BEC infected with *S. aureus*. Data in this work indicate that the relative abundance of phospho-GSK3α was higher than that of phospho-GSK3β during the infection of BEC with viable *S. aureus* (Figures 3A,B), as we have previously reported (18). The predominance of phospho-GSK3α over phospho-GSK3β in our system suggests specific roles for each GSK3 isoform during the inflammatory response triggered by *S. aureus*. At initial stages of infection, a gradually decreasing fraction of GSK3α and in minor proportion GSK3β remains activated, implying an indirect correlation between the amount of GSK3α in its active form and the expression of IL-8. In line with this thought, we found that complete GSK3α inhibition at 120 min post infection led to a substantial reduction of the NF-κB-CBP complex compared with the abundance of this complex observed at 40 min post infection (Figure 6A) and a high increase in CREB-CBP interaction (Figure 6B). Furthermore, the IL-8 expression increased and remained at high levels after 6 h when Akt was inhibited to avoid GSK3α inhibition (Figure 8A). These data indicate that GSK3α activity is required to maintain IL-8 expression at longer infection periods and that GSK3α inhibition was related to a loss of NF-κB transcriptional activity and the activation of CREB.

Another important issue derived from our results is how GSK3α represses the phosphorylation of CREB at Ser133 because GSK3 is unable to phosphorylate CREB in this residue. An intriguing possibility is that GSK3 constitutive activity controls an unidentified CREB kinase. In support of this notion, GSK3α was identified in rat cerebral cortical cells as the responsible isoform that represses CREB transcriptional activity (58) and reduces the affinity of Akt against different substrates by phosphorylating Akt at Thr312 (59). In addition, siRNA silencing of the GSK3α gene expression enhanced phospho-CREB-Ser133 and CRE transcriptional activity (59). In agreement, our evidence shows that Akt activity was critical to increase phospho-CREB-Ser133, which suggests that GSK3α inhibition promotes CREB phosphorylation at Ser133 by an Akt-dependent mechanism. Importantly, GSK3α is now known as a novel CREB-target gene that promotes the viability of cancer cells and NF-κB-dependent gene transcription of TERT (60), indicating a reciprocal regulation between CREB and GSK3α. Further investigation on the mechanisms of this reciprocal regulation deserves consideration in the context of the bacteria-activated inflammatory response.

In conclusion, during the first 60 min of BEC infection by *S. aureus*, GSK3α activity maintains its inhibitory influence on CREB phosphorylation at Ser133, limiting the formation of CREB-CBP complex and leaving CBP free to interact with NF-κB. This mechanism promotes a pro-inflammatory environment because of the IL-8 expression. If the stimulus persists for 120 min, GSK3α/β becomes fully phosphorylated by Akt, which indirectly inhibits NF-κB transcriptional activity. The inhibition of GSK3α, and in minor proportion GSK3β, causes an increase in phospho-CREB-Ser133, which is now able to compete for CBP to form the transcriptionally active CREB-CBP complex. Such a molecular switch markedly reduces the expression of pro-inflammatory chemokine IL-8 and stimulates the expression of immune-suppressive and -tolerogenic cytokine IL-10. More experiments will be undoubtedly needed to clarify the specific mechanistic details of the differential actions of GSK3α on the regulation of the inflammatory response caused by pathogenic bacteria.

## Author Contributions

OS-G contributed with ideas of experimental design, performed 90% of the experiments, analyzed results, and wrote the first draft; RR-M performed 5% of the experiments; MM-P, performed 5% of the experiments; AB-P, JV-A, and JA-G critical review and corrections of this manuscript; VB-A central idea of the research, wrote this manuscript, and financially supported the research.

## Conflict of Interest Statement

The authors declare that the research was conducted in the absence of any commercial or financial relationships that could be construed as a potential conflict of interest.
