# Supplementary figures and images for: Glycogen Synthase Kinase 3α Is the Main Isoform That Regulates the Transcription Factors Nuclear Factor-Kappa B and cAMP Response Element Binding in Bovine Endothelial Cells Infected with Staphylococcus aureus
Source: Front Immunol. 2018 Jan 29;9:92. doi: 10.3389/fimmu.2018.00092 (PMC5796901; doi:10.3389/fimmu.2018.00092)

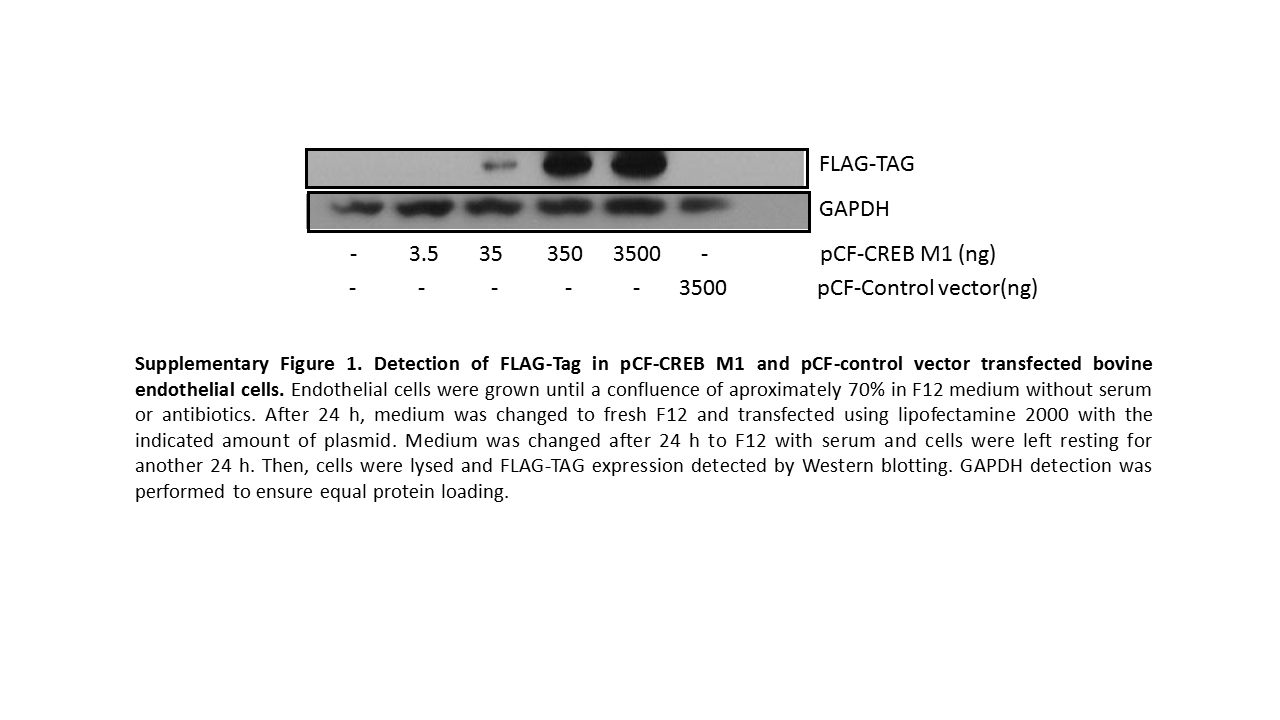

Supplement: Supplementary file 1 [file Image_1.TIF]
